# Supplementary material for: The Arabidopsis leaf quantitative atlas: a cellular and subcellular mapping through unified data integration
Source: Quant Plant Biol. 2024 Feb 29;5:e2. doi: 10.1017/qpb.2024.1 (PMC10988163; doi:10.1017/qpb.2024.1)
Supplement: Tolleter et al. supplementary material 1 — Tolleter et al. supplementary material [file S2632882824000018sup001.docx]

**Procedure used to convert metabolite abundance in various units into concentrations in µM.**

We used as input the quantitative data stored in ChloroKB. This file can be downloaded from the web site (see Excel version in Table S3) and contains all the information required to evaluate the origin of the data (metabolite name, chemical identifiers, plant developmental state, biological material used for the quantification, plant species, experimental growth conditions, comments). A python script (available on zenodo (<https://doi.org/10.5281/zenodo.10410541>) was written to convert concentrations as found in the literature in specific units into a single one (µM).

1. When metabolites concentrations are expressed in µM in the original paper they were kept as such, *i.e* conversion was already done by the authors using their own conversion factors.
2. pH units were converted into molar concentration of protons
3. Data related to other tissues than leaf (hypocotyl, inflorescence, root, seed) were not converted into molar concentrations as volumetric cellular parameters are still unknown for these tissues.
4. The various remaining units for whole leaf extraction (*e.g*. µg/mm^2 etc.) were converted into nanomol/g FW if not already provided with this unit in the original papers. To simplify the conversions, the same conversion factors were applied to the following different cases of “source biological material”: Leaf, mature leaf, old leaf, young leaf, rosette, seedling, protoplast, and for the “compartments” where the metabolites of interest were quantified, *i.e*. « Whole leaf » or « Whole cell ». When non-aqueous fractionation method was used the metabolites amounts in the different fractions (chloroplast, vacuole, cytosol) are expressed relatively to the initial plant leaf material (in nanomol /g FW) and did not require conversion at this step. Depending on the units found in the original works different conversions had to be applied (see Table S5 (named variable_conversion_table in the python script and python script in the supplementary material). The formulas used and the converting factors are based on the experimental data detailed and referenced in Supplementary Table S1.1.
5. Conversion of units in nanomol/g FW (whole leaf, whole cell, seedling) into µM concentrations.

To convert metabolites amount into molar concentration when starting material was an entire leaf, the localisation of the metabolites in the cell had to be set using knowledge or assumptions. The different leaf cells display various metabolic activities and obviously different metabolite concentrations. To simply calculations we neglected the contribution of epidermal and vein cells. As 85% of the liquid volume of the leaf is contributed by mesophyll cells, neglecting specific contribution of epidermal cells and vein cells will lead to acceptable errors in the estimation of metabolite concentrations as long as the aim is to provide orders of magnitude rather that exact values. In addition, previous work showed that spongy and palisade mesophyll cell have the same metabolism ([Outlaw *et al.*, 1976](#_ENREF_2)). To calculate automatically the concentration from ChloroKB quantative_data.csv export file a “curated localization“ column was added. This specification takes into account both the biological material used (*e.g*. leaf or isolated chloroplasts or isolated vacuole etc. ) and the knowledge about the cellular localization of the metabolite considered. Several examples are described below to explain how we proceeded. If a metabolite has been quantified,for example, in isolated chloroplasts and is known to be in the stroma, the curated localization will be “stroma”. If, in another study the same metabolite has been quantified in the entire leaf the curated localization will also be “stroma” if we know this metabolite is only localized in this subcellular compartment (a typical example is phosphoglycolate). If the metabolite is present in several compartments (*e.g*. glycolate) ChloroKB reconstructed network was used to check the different possible localizations: for the present example, the curated localization is “ cytosol, peroxisome, stroma (photosynthetic cells)”. In this example and the other cases where the metabolite is present in several compartments and quantified in leaf or whole cell we had to assumed the same concentration in the different compartments. Thus, the amount of metabolite in nanomol per g LFW was divided by the sum of the volume of each compartment in µL per g LFW.

In some cases the curated localization remains hypothetical. For example we could not find data showing presence of oxaloacetate in the vacuole. The curated localization chosen was “cytoplasm excluding vacuole (photosynthetic cells)”. When new data is issued this curated localization will be changed and the computation will be automatically modified after each new update of ChloroKB.

The python script relies on data extracted from Table S1.1 and included in the three sheets of Table S4. Table S4.1, named Variable_conversion_table in the code was used as a first conversion step for example to convert quantities expressed “per mg chlorophyll” into “per g LFW”; Table S4.2 (included in the Python script) was used to convert concentrations expressed in one of the 17 units founds in the literature into nanomol/g FW; Table S4.3 (named localisation_conversion_table in the python script) contains the conversion factors deduced from volumetric data (*i.e* conversion factors in lines #943 to #994 in Table S1.1); Table S4.4 (named Localisation_conversion_table_isolated_organelles in the python script) was specifically used for conversion of concentration measured with isolated organelles (the conversion factors are from Table S1.1, lines #998 to #1004).

For quantitative data obtained with other plants than Arabidopsis we used as a first approximation the conversion factors established for Arabidopsis. Comparison of volumetric data published by ([Winter *et al.*, 1994](#_ENREF_3)) for *Spinacia oleracea* and ([Leidreiter *et al.*, 1995](#_ENREF_1)) for *Solanum tuberosum* with the data we provide here for Arabidopsis suggests that this simplification is reasonable as long as orders of magnitude are considered.

The results of this data treatment are displayed in Supplementary Table S5 and the concentrations in µM are displayed in the last columns. The name of the metabolites is in the first column and is duplicated in the last column to facilitate reading and use.

**Precision for molecules isomers**: Some metabolites are present under different isomeric forms in spontaneous equilibrium *in vivo* (*e.g*. α-glucose and β-glucose). The concentration indicated in Table S6 for such isomers represent the sum of the concentration of the different isomeric forms. The abundance of each specific isomer in the cellular environment could be calculated based on the equilibrium constant.

References:

**Leidreiter K, Kruse A, Heineke D, Robinson DG, Heldt HW. 1995.** Subcellular volumes and metabolite concentrations in potato (solanum-tuberosum cv desiree) leaves. *Botanica Acta* **108**(5): 439-444.

**Outlaw WH, Schmuck CL, Tolbert NE. 1976.** Photosynthetic carbon metabolism in palisade parenchyma and spongy parenchyma of vicia-faba-l. *Plant Physiology* **58**(2): 186-189.

**Winter H, Robinson DG, Heldt HW. 1994.** Subcellular volumes and metabolite concentrations in spinach leaves. *Planta* **193**: 530-535.
