# Supplementary material for: The Arabidopsis leaf quantitative atlas: a cellular and subcellular mapping through unified data integration
Source: Quant Plant Biol. 2024 Feb 29;5:e2. doi: 10.1017/qpb.2024.1 (PMC10988163; doi:10.1017/qpb.2024.1)
Supplement: Tolleter et al. supplementary material 2 — Tolleter et al. supplementary material [file S2632882824000018sup002.pptx]

## Slide 1
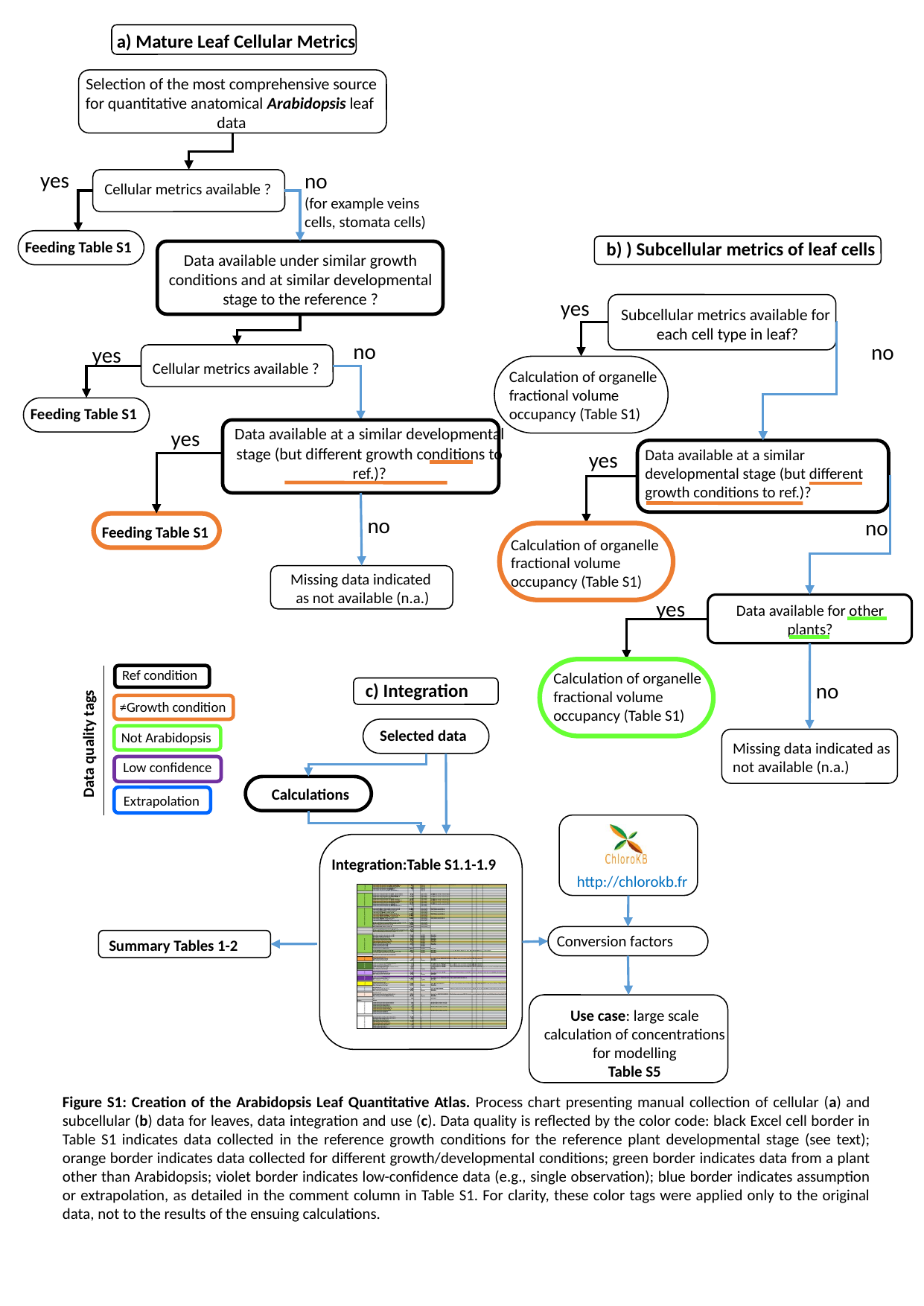

a) Mature Leaf Cellular Metrics
Selection of the most comprehensive source for quantitative anatomical Arabidopsis leaf
data
yes
no
(for example veins cells, stomata cells)
Cellular metrics available ?
Feeding Table S1
b) ) Subcellular metrics of leaf cells
Data available under similar growth conditions and at similar developmental stage to the reference ?
yes
Subcellular metrics available for
each cell type in leaf?
no
no
yes
Cellular metrics available ?
Calculation of organelle fractional volume occupancy (Table S1)
Feeding Table S1
Data available at a similar developmental stage (but different growth conditions to ref.)?
yes
Data available at a similar developmental stage (but different growth conditions to ref.)?
yes
no
no
Feeding Table S1
Calculation of organelle fractional volume occupancy (Table S1)
Missing data indicated
 as not available (n.a.)
yes
Data available for other plants?
Ref condition
Calculation of organelle fractional volume occupancy (Table S1)
no
c) Integration
≠Growth condition
Not Arabidopsis
Low confidence
Extrapolation
Selected data
Data quality tags
Missing data indicated as
not available (n.a.)
Calculations
http://chlorokb.fr
 Integration:Table S1.1-1.9
Conversion factors
 Summary Tables 1-2
 Use case: large scale
calculation of concentrations
for modelling
Table S5
Figure S1: Creation of the Arabidopsis Leaf Quantitative Atlas. Process chart presenting manual collection of cellular (a) and subcellular (b) data for leaves, data integration and use (c). Data quality is reflected by the color code: black Excel cell border in Table S1 indicates data collected in the reference growth conditions for the reference plant developmental stage (see text); orange border indicates data collected for different growth/developmental conditions; green border indicates data from a plant other than Arabidopsis; violet border indicates low-confidence data (e.g., single observation); blue border indicates assumption or extrapolation, as detailed in the comment column in Table S1. For clarity, these color tags were applied only to the original data, not to the results of the ensuing calculations.
