# Supplementary material for: The Arabidopsis leaf quantitative atlas: a cellular and subcellular mapping through unified data integration
Source: Quant Plant Biol. 2024 Feb 29;5:e2. doi: 10.1017/qpb.2024.1 (PMC10988163; doi:10.1017/qpb.2024.1)
Supplement: Tolleter et al. supplementary material 3 — Tolleter et al. supplementary material [file S2632882824000018sup003.pptx]

## Slide 1
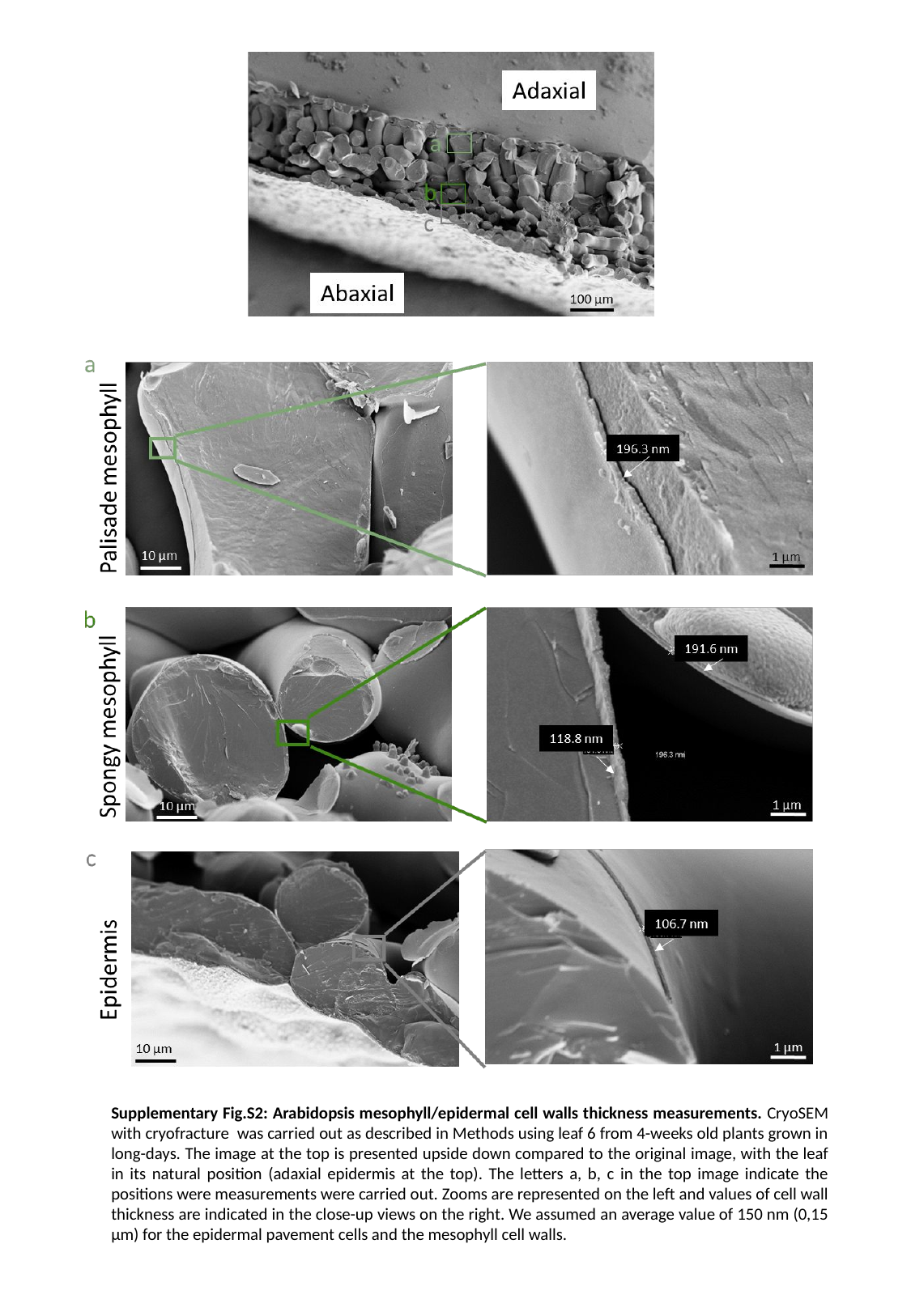

Supplementary Fig.S2: Arabidopsis mesophyll/epidermal cell walls thickness measurements. CryoSEM with cryofracture was carried out as described in Methods using leaf 6 from 4-weeks old plants grown in long-days. The image at the top is presented upside down compared to the original image, with the leaf in its natural position (adaxial epidermis at the top). The letters a, b, c in the top image indicate the positions were measurements were carried out. Zooms are represented on the left and values of cell wall thickness are indicated in the close-up views on the right. We assumed an average value of 150 nm (0,15 µm) for the epidermal pavement cells and the mesophyll cell walls.
