# Supplementary material for: The Arabidopsis leaf quantitative atlas: a cellular and subcellular mapping through unified data integration
Source: Quant Plant Biol. 2024 Feb 29;5:e2. doi: 10.1017/qpb.2024.1 (PMC10988163; doi:10.1017/qpb.2024.1)
Supplement: Tolleter et al. supplementary material 4 — Tolleter et al. supplementary material [file S2632882824000018sup004.pptx]

## Slide 1
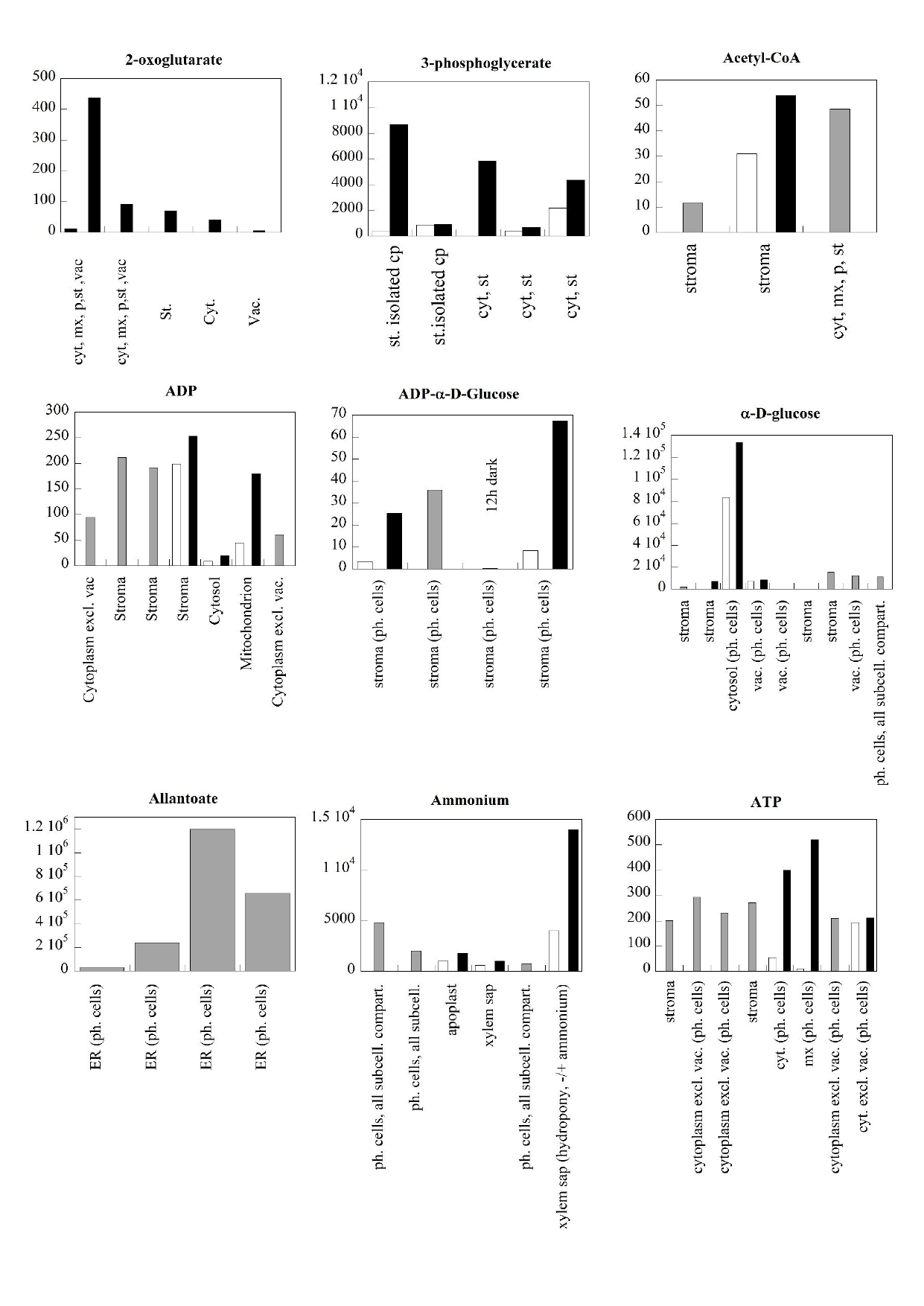

## Slide 2
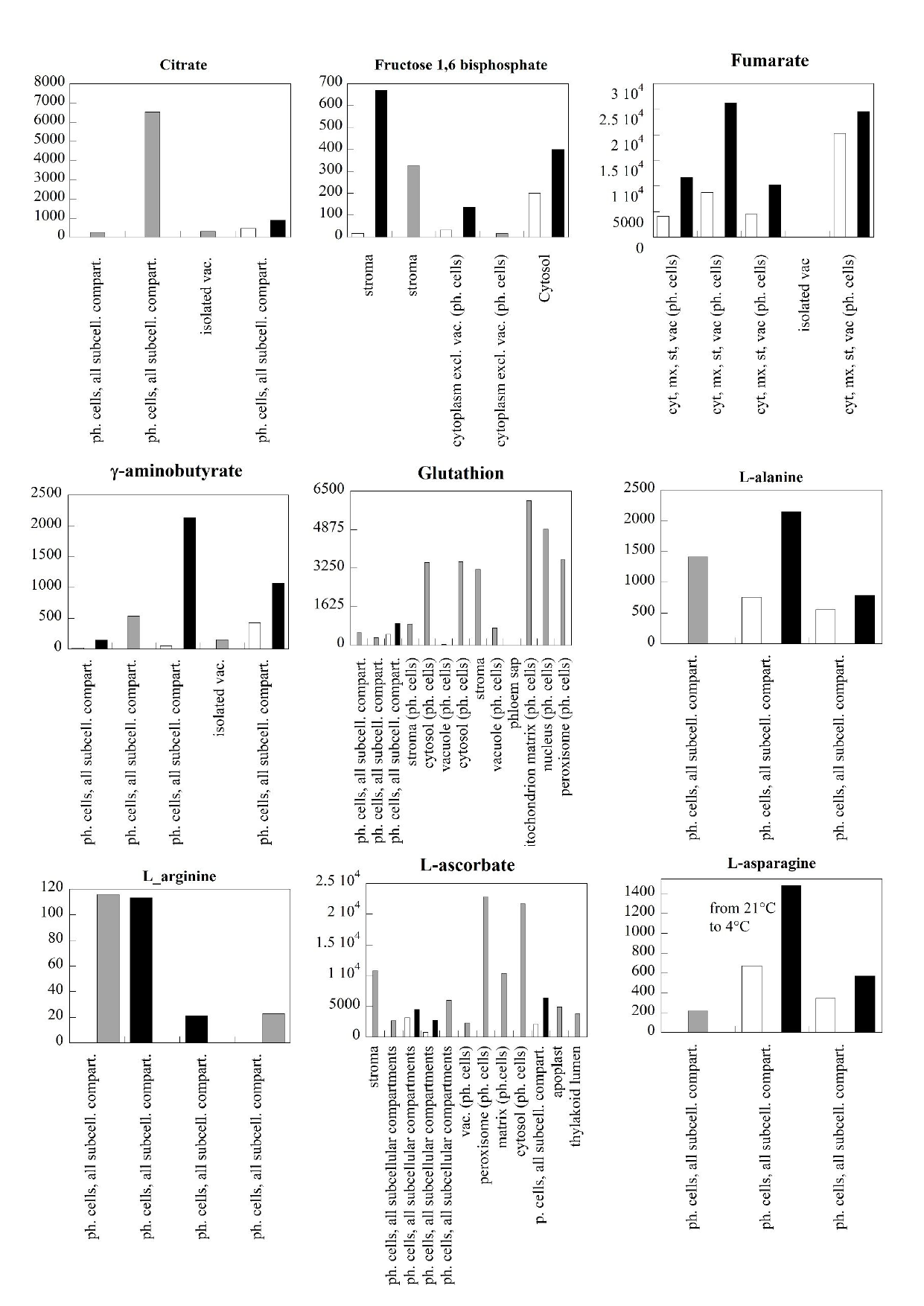

## Slide 3
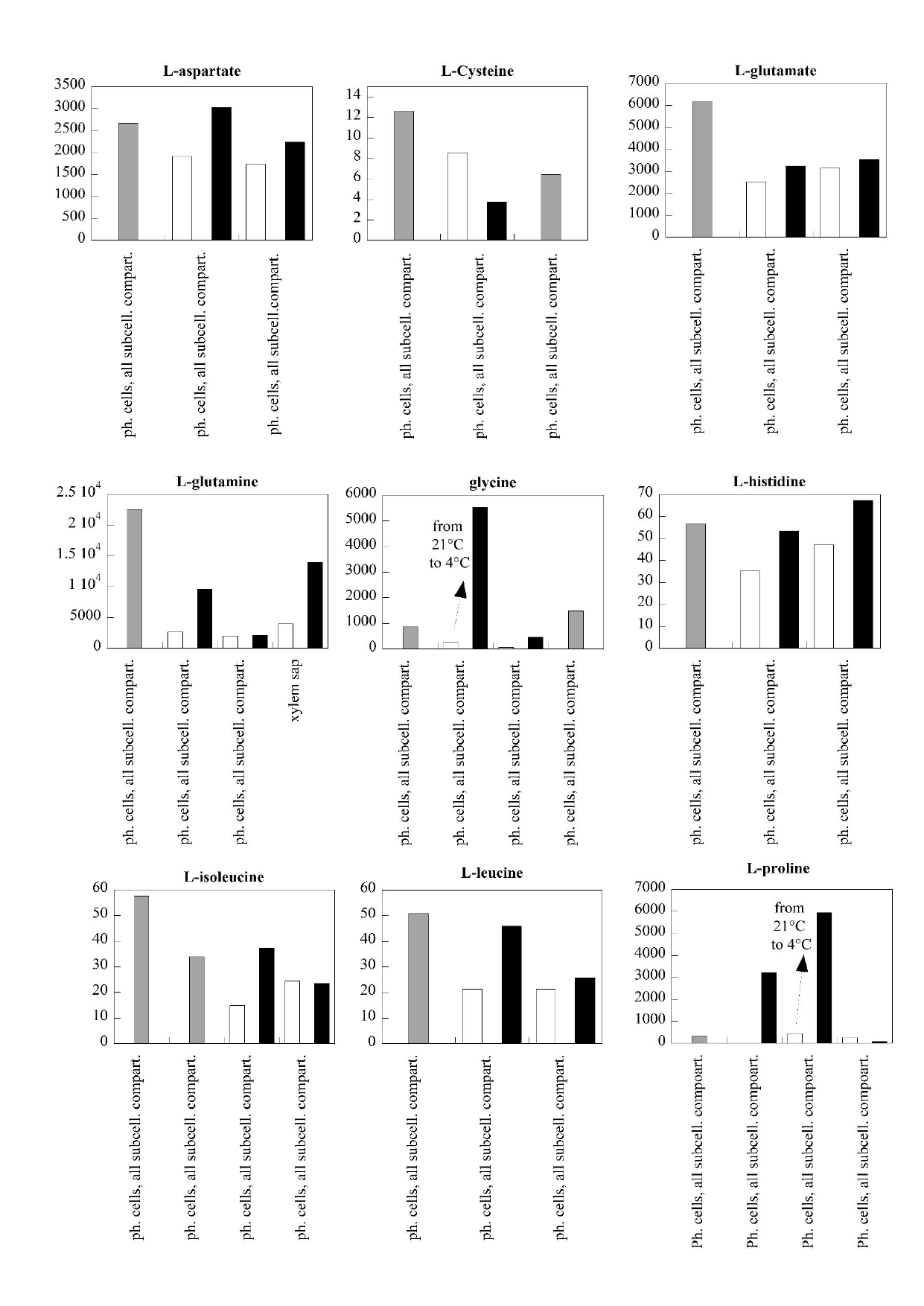

## Slide 4
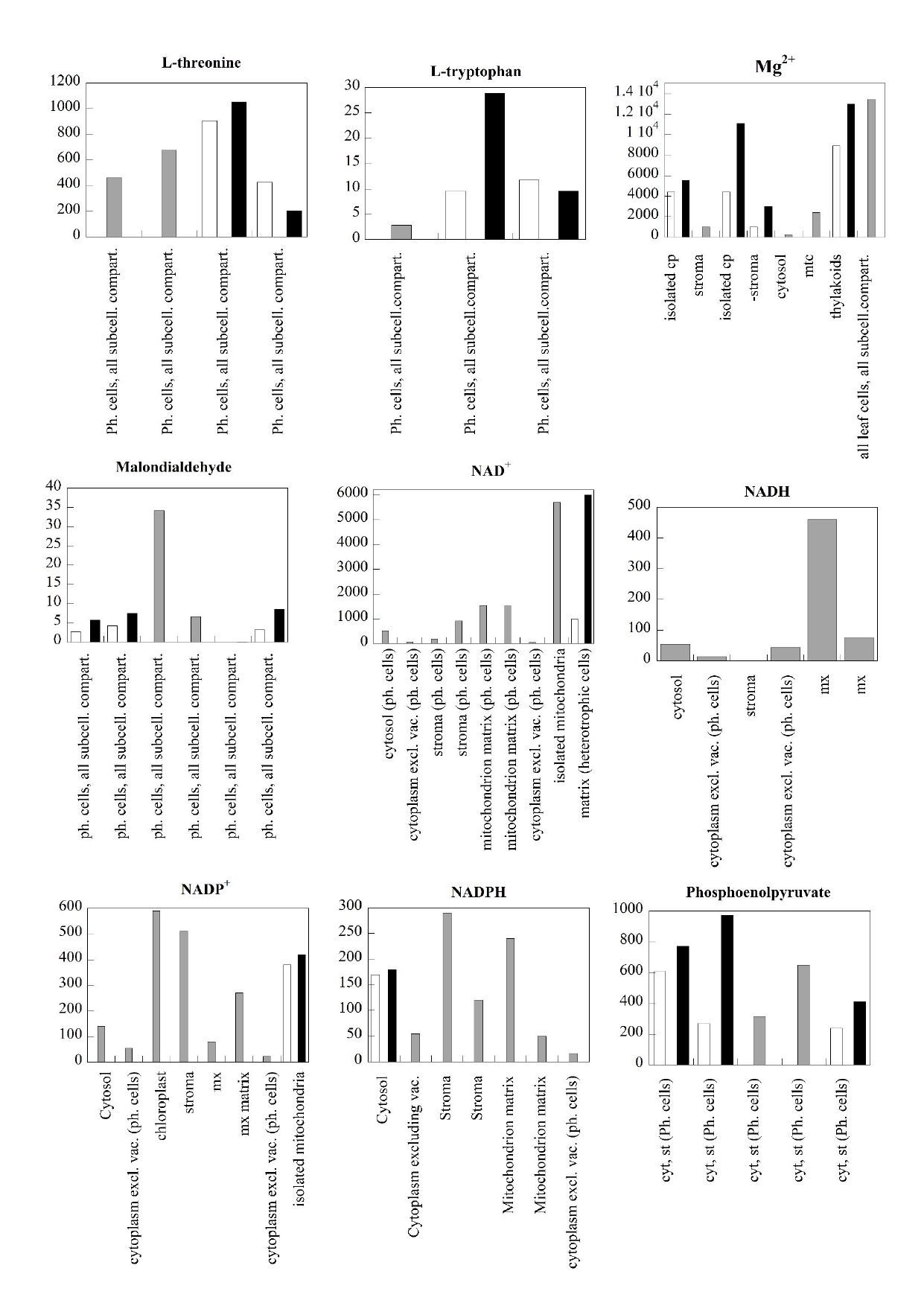

## Slide 5
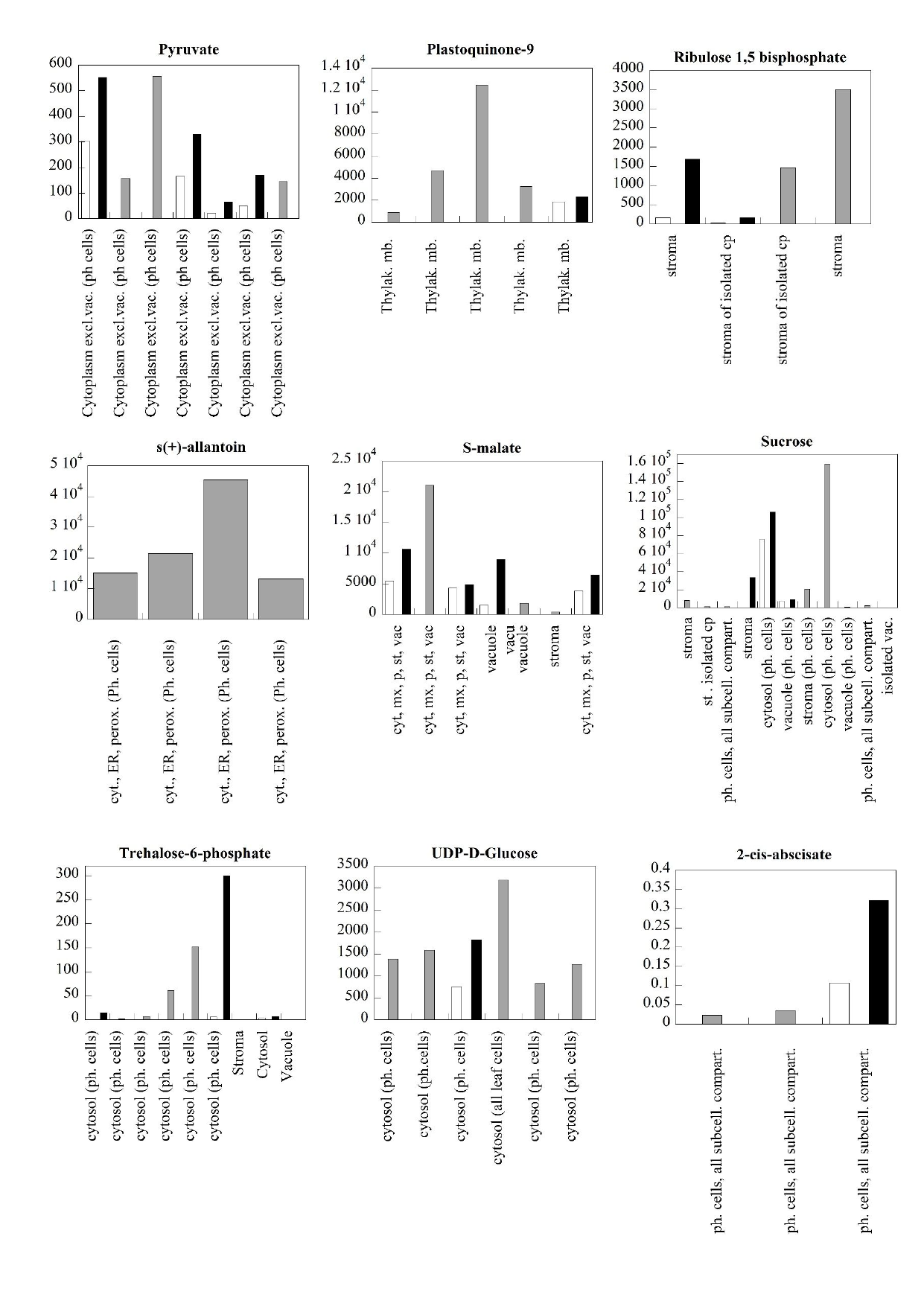

## Slide 6
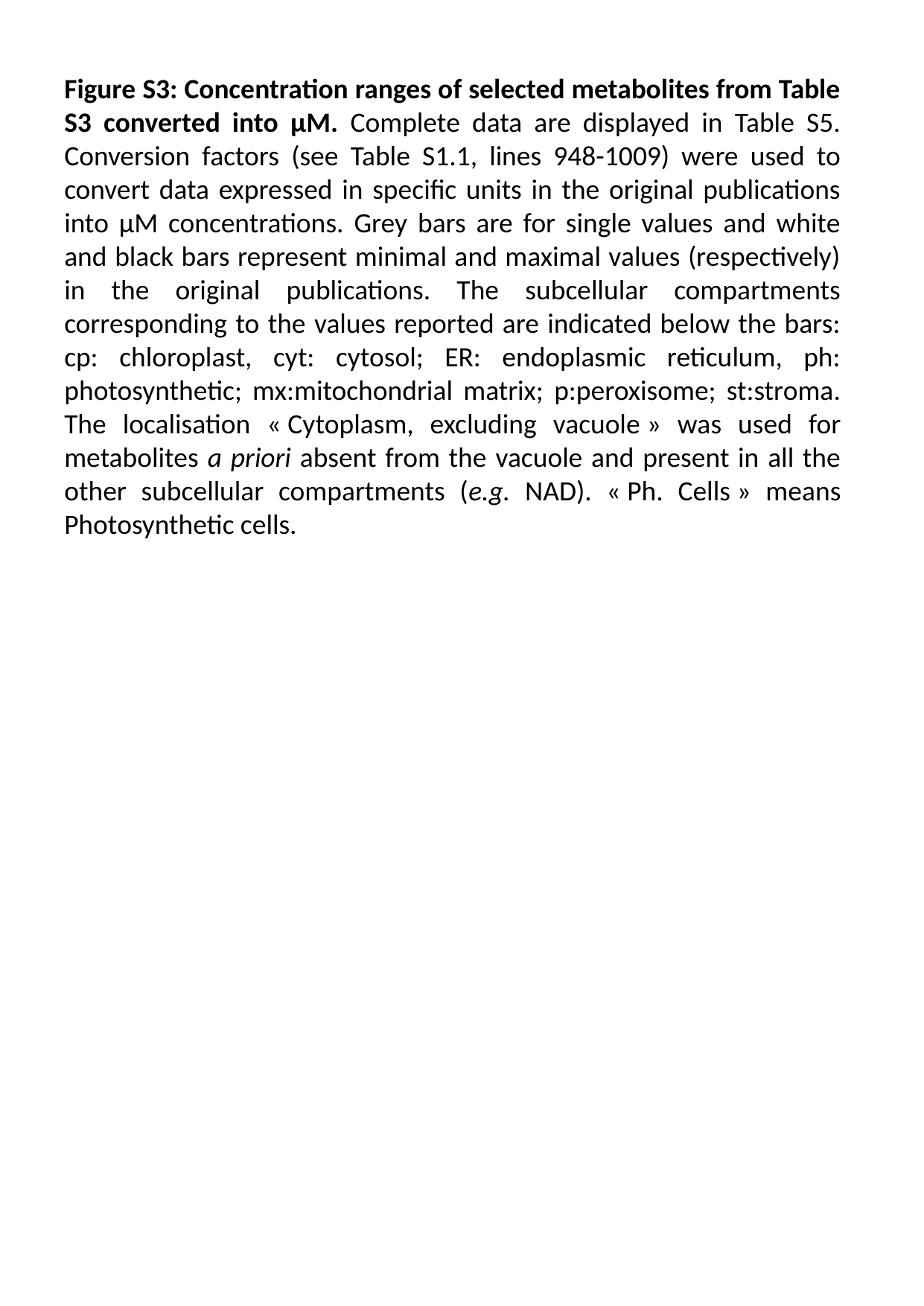

Figure S3: Concentration ranges of selected metabolites from Table S3 converted into µM. Complete data are displayed in Table S5. Conversion factors (see Table S1.1, lines 948-1009) were used to convert data expressed in specific units in the original publications into µM concentrations. Grey bars are for single values and white and black bars represent minimal and maximal values (respectively) in the original publications. The subcellular compartments corresponding to the values reported are indicated below the bars: cp: chloroplast, cyt: cytosol; ER: endoplasmic reticulum, ph: photosynthetic; mx:mitochondrial matrix; p:peroxisome; st:stroma. The localisation « Cytoplasm, excluding vacuole » was used for metabolites a priori absent from the vacuole and present in all the other subcellular compartments (e.g. NAD). « Ph. Cells » means Photosynthetic cells.
